# Who, What, Where, When, Why, and How (5W1H)-Based Education of Informed Consent for Intravenous Thrombolysis in Acute Ischemic Stroke

**DOI:** 10.7759/cureus.101137

**Published:** 2026-01-09

**Authors:** Yan Wang

**Affiliations:** 1 Emergency and Disaster Medicine, The Seventh Affiliated Hospital of Sun Yat-sen University, Shenzhen, CHN

**Keywords:** 5w1h, acute ischemic stroke, informed consent, intravenous thrmbolysis, medical education

## Abstract

Intravenous thrombolysis (IVT) is an effective approach for restoring cerebral blood flow in acute ischemic stroke (AIS). Its benefit is highly time-dependent, and its safety considerations must not be overlooked. Rapid and efficient acquisition of informed consent is critical. Therefore, targeted training in informed consent competencies for healthcare personnel working in the stroke green channel is warranted. We introduced the 5W1H model into informed consent education for IVT in AIS, focusing on the Why (rationale), Who (actors and recipients), What (content), When (timing), Where (location), and How (methods). We employed a teaching approach that combined theoretical lectures, cohort-based learning, and scenario simulations. We aimed to improve communication skills among stroke green channel staff, increase the efficiency of obtaining informed consent for IVT, and achieve more comprehensive and effective clinician-patient communication.

## Introduction

In recent years, stroke has become the leading cause of death among adults in China, with acute ischemic stroke (AIS) accounting for the majority of incident stroke in the country [1,2]. Intravenous thrombolysis (IVT) is an important and effective method for restoring cerebral perfusion in AIS. However, the conventional therapeutic time window for IVT is only 3-4.5 hours, and the benefit of IVT is highly time-dependent and diminishes with delay [3]. Although imaging-based issue selection for patients beyond the conventional window has received increasing attention, eligible patients still require urgent reperfusion therapy. Furthermore, IVT carries safety risks, including hemorrhagic transformation, reperfusion injury, systemic bleeding, allergic reactions, and vasogenic cerebral edema. For these reasons, rapid and effective acquisition of informed consent for IVT is particularly critical. Patients’ autonomy should be respected in clinical practice [4]. Because of the abrupt onset of AIS, the frequently urgent need for IVT, and various objective factors, obtaining informed consent rapidly and effectively can be challenging. Accordingly, targeted training in informed consent competencies for staff working in the stroke green channel is of critical importance. The stroke green channel refers to a priority pathway established by hospitals for patients with acute stroke upon their arrival, ensuring they receive prompt diagnosis, timely treatment, and continuous monitoring. This measure aims to reduce waiting times for patients with stroke and restore cerebral blood flow as early as possible, thereby minimizing brain damage and improving prognosis. This study introduces the 5W1H model into the design of informed consent education for intravenous thrombolysis in AIS and conducts teaching through this conceptual framework. It aims to improve communication skills among stroke green channel staff, increase the efficiency of obtaining informed consent for IVT, and achieve more comprehensive and effective clinician-patient communication.

## Technical report

The “5W1H” model

The 5W analytic method was first proposed by Lasswell in 1932. Through continued application and refinement, it has evolved into the 5W1H framework-Why (reason), Who (subjects and recipients), What (content), When (timing), Where (location), and How (methods) [5,6]. This study aims to introduce the 5W1H model into the informed consent education for IVT in AIS. This approach seeks to maximize the "patient-centered" principle and improve the operational efficiency of the stroke green channel (Table 1).

**Table 1:** Introduction of the “5W1H” model into the informed consent education for IVT in AIS 5W1H: who-what-where-when-why-and-how; IVT: intravenous thrombolysis; AIS: acute ischemic stroke.

“5W1H”		Components
Why		The health risks posed by AIS
		The time-dependent nature of IVT
		The role of IVT in salvaging the ischemic penumbra and improving neurological deficits
Who	Agents	The treating IVT physician
		Other personnel along the stroke green channel, including prehospital emergency medical staff, triage nurses, and emergency department clinicians and nurses.
	Recipients	The patients and their legally authorized representatives
What		Objective information grounded in the pathophysiology of AIS
		Clinical risk
		Information that is in accordance with individualized communication principles
When		Initial contact with the patient
		During history taking
		While performing the neurological examination
		During cranial imaging
How		Information layering
		Use of plain language
		Integration of group education with individual informed consent
		Clinician–patient empathy

Why (Reason)

AIS is characterized by high incidence, high recurrence, high disability, and high mortality, imposing a heavy burden on patients, families, and society [7]. IVT is one of the most effective methods for restoring cerebral blood flow in AIS [3]. However, the conventional time window is only 3-4.5 hours [3], making the opportunity for treatment fleeting. Thrombolytic efficacy is markedly time-dependent, with earlier intervention yielding better outcomes. Although tissue-based selection of patients beyond the conventional time window has received increasing attention, eligible patients within the standard window still require urgent thrombolytic treatment [8]. In clinical education, instructors should train learners to help patients who meet IVT criteria and their legal representatives rapidly understand the health risks posed by AIS, the time-dependent nature of IVT, and its role in salvaging the ischemic penumbra and improving neurological deficits, thereby fostering a shared commitment to the principle that “time is brain.”

Who (Agents and Recipients)

The primary agents of clinician-patient communication are the healthcare professionals [9]. Decisions regarding IVT rest with the treating physician. Therefore, the process of obtaining informed consent should be led by that physician. Meanwhile, trainees should be guided to appreciate the supporting communication roles of other personnel along the stroke green channel, including prehospital emergency medical staff, triage nurses, and emergency department clinicians and nurses. These team members can provide an initial explanation to patients and their legal representatives about the basic logic of the possibility of acute stroke, the rationale for activating the green channel pathway, and subsequent IVT evaluation. This activity is not only a component of clinical workflow but also an important educational scenario that helps trainees learn how interprofessional collaboration can improve patients’ understanding of the disease and acceptance of treatment, thereby laying the foundation for the IVT physician’s subsequent, more detailed communication.

The recipients of clinician-patient communication are the patients and their legally authorized representatives. Autonomy is a fundamental right of adults who possess decision-making capacity. Decision-making capacity requires the cognitive and emotional faculties necessary for informed choice [10]. Factors that may impair capacity include aphasia, limited expressive or receptive communication, impaired comprehension, low literacy or educational attainment, impaired judgment, lack of legal competence, disorders of consciousness and so forth [11]. In clinical teaching, trainees should be guided to systematically appreciate the complexity of patients and their legal representatives, to perform rapid assessments for communication barriers, and to flexibly apply communication strategies such as conveying information through a legal representative, using visual aids to support explanations, or modifying language and phrasing. Such training helps ensure that informed consent is effectively obtained in both educational and clinical settings while respecting patients’ autonomy.

What (Content)

In the informed-consent process for AIS treatment, clinicians must identify appropriate therapeutic options, present the available treatments and alternatives, and elicit the patients’ values, preferences, and concerns related to therapy and outcomes [9]. In clinical teaching, instructors should focus on guiding trainees to master three key domains: (1) Firstly, physicians should provide objective information grounded in the pathophysiology of AIS [12]. Trainees should be taught to clearly explain the mechanism of IVT: rapid recanalization of the occluded artery can salvage threatened neurons within the ischemic penumbra, thereby improving neurological function and reducing the risk of disability. At the same time, it must be communicated that neurons within the core infarct have already undergone irreversible injury, and consequently, preexisting deficits may not completely resolve after treatment; (2) Secondly, physicians should incorporate clinical risk counseling into the informed-consent process. Instructors should train learners to communicate to patients and their legal representatives the potential risks and uncertainties of IVT, including failure to achieve thrombus dissolution, thrombus fragmentation with distal embolization, vessel reocclusion, and complications such as intracranial hemorrhage, systemic bleeds, allergic reactions and so on; and (3) Thirdly, physicians should apply individualized communication principles throughout the informed-consent process. Instructors should help trainees recognize the multiple challenges that complicate consent discussions, including the intrinsic complexity of the disease, variability in patients’ baseline knowledge and decision-making capacity, the time-critical constraints of emergency care, and concerns about post-stroke quality of life [13]. Patient-specific factors, including age, time from symptom onset, subtype of stroke, severity of neurologic deficit, status of the occluded vessel and collateral circulation, comorbidities, and individuals’ risk tolerance, which differ substantially and influence the balance of benefits and harms [14]. Therefore, instructors should emphasize teaching learners to tailor both the content and delivery of information to the individual clinical context, combining accurate, evidence-based disclosure with humane, empathic communication to help patients or their surrogates make well-informed, value-concordant decisions.

When (Timing)

Informed consent should always allow sufficient time to discuss all possible complications and treatment options [15]. However, there is often severe time pressure in the clinical setting of IVT for AIS. A Dutch study found that most clinicians reported needing to obtain patients’ consent within one minute [16]. Communication under such extreme time constraints readily produces conflicts among legal, ethical, and clinical time considerations [16]: the law requires adequacy of disclosure, ethics emphasizes respect for patient autonomy, and clinical practice urgently needs to preserve the treatment time window. 

Therefore, beyond continuously improving trainees’ communication skills, instructors should cultivate a “time-management” mindset and promote optimization of the green channel workflow to increase informed‑consent efficiency. Concretely, trainees can be trained to seize “pre‑diagnosis” communication opportunities: explain the basic principles of IVT for AIS, the expected benefits, the major risks, and available alternatives in a stepwise, layered manner after initial contact with the patient, during history taking, while performing the neurological examination, and during cranial imaging. This proactive, staged‑communication strategy not only relieves the time pressure of concentrated post‑diagnosis disclosure, but also gives patients (or their surrogates) more time to consider and ask questions, thereby respecting patients’ autonomy while preserving treatment timeliness.

Where (Location)

In teaching informed consent for IVT in AIS, the proactive, front‑loaded communication strategy not only reflects scientific planning of communication timing but also involves optimizing choice of communication location. Studies have showed that informed consent should be obtained in a relatively appropriate environment to ensure effective information transfer and high‑quality clinician-patient interaction [15]. Therefore, instructors should guide trainees to recognize the importance of environmental factors for consent quality, attend to issues such as ambient noise and privacy protection, and avoid conducting key treatment‑decision conversations in noisy, crowded, or highly distracting areas whenever possible [11]. Instructors can guide trainees to acknowledge constraints and offer acceptable fallback options in crowded emergency department settings. They can also consider implementing pragmatic mitigation steps (privacy screen, designating quiet corner, limiting bystanders, assigning a single communicator).

How (Methods)

Multiple studies indicate that several patient-related factors commonly hinder effective informed consent in clinical practice, including limited health literacy, lack of willingness to participate, difficulty understanding medical terminology, inadequate comprehension of treatment options, and absence of family involvement [17-19]. Therefore, in the teaching process, instructors should guide trainees to master the following communication skills: (1) Information layering: During informed consent, clinicians can first convey core information such as treatment urgency and key risks, and then gradually delve into the details; (2) Use of plain language: Clinicians should explain in simple, clear, and easy-to-understand terms what stroke is, why IVT is used, the key risks of IVT, and why a prompt decision is necessary. For example, physicians can combine text and images to enhance patient comprehension, rendering the technical term “recombinant tissue plasminogen activator” into everyday language such as “a clot-dissolving, vessel-opening medicine”. Instructors can encourage trainees to use metaphors. For example, physicians can compare AIS to “crops wilting and drying from lack of water”, IVT to “emergency repair of a blocked irrigation channel”, and the time window to a “golden rescue period” in order to improve patients’ understanding; (3) Integration of group education with individual informed consent: Clinicians can play brief educational videos about IVT for AIS to relieve patients’ stress and sense of urgency and to improve their acceptance and cooperation. Meanwhile, communication strategies should be flexibly adjusted and tailored to each patient’s individual circumstances; and (4) Clinician-patient empathy: The ability to express empathy is a core competence for healthcare professionals and can provide very important psychological support to patients [20]. Instructors should reinforce trainees’ empathy training, including identifying patients’ emotions such as fear, hesitation and so on, responding to patients’ emotional needs, and using appropriate body language such as leaning forward slightly, making eye contact and so on.

Implementation of the teaching approach

Informed consent for IVT in AIS is highly practical, while traditional didactic teaching cannot reproduce the complexity and urgency of the clinical setting. Therefore, educators should shift their teaching philosophy from "knowledge transmission" to "competency building", so that learners can not only master the workflow but also complete informed consent effectively, humanely, correctly, and quickly under real clinical pressure. In instructional design, teachers can create multi-role, end-to-end, high-fidelity scenarios.

This study employed a structured teaching approach aimed at providing a comprehensive and reproducible framework to enhance the informed consent process for IVT in AIS through the application of the 5W1H model. The training targeted healthcare professionals with over one year of clinical experience directly involved in the management of AIS, including emergency department physicians, neurologists, nurses, and related medical staff. The training integrated theoretical lectures, bedside learning, and scenario simulations. In the theoretical lecture segment, the focus was on introducing the theoretical knowledge of the 5W1H model and its application in the informed consent process. The bedside learning component emphasized guiding participants through clinical observation to learn communication skills from instructors. During the scenario simulation phase, participants engaged in role-playing exercises to simulate the informed consent process, practicing communication using the 5W1H framework. Instructors assessed participants' performance in these exercises and evaluated their ability to effectively apply the 5W1H model in informed consent discussions, providing real-time feedback and guidance.

This structured teaching approach aimed to equip healthcare professionals with the necessary skills to enhance the informed consent process in AIS management, thereby improving patient understanding and decision-making. Specific strategies included developing a standardized case library and increasing immersive training modalities such as scenario-based simulation, allowing learners to take turns playing patients, family members, and various roles within the stroke green channel pathway. This approach can enhance learners’ engagement, participation, and practical skills, deepen perspective-taking, and optimizes team collaboration, so that learners not only acquire the skills for IVT informed consent but also develop adaptability to complex clinical communication situations. Besides, this teaching strategy enabled instructors to identify learners’ problems in real time and provide individualized guidance.

## Discussion

Informed consent is a core principle of bioethics and binding in both the moral and legal respects [21,22]. Its purpose is not to mitigate or avoid risk, but is fundamentally grounded in protecting patients’ interests and fostering a more harmonious doctor-patient relationship [23,24]. Informed consent is the cornerstone of contemporary medical practice. Consent given without adequate information is likely to provoke doctor-patient conflict and may even lead to medical disputes. The efficiency of informed consent for IVT in AIS has a positive impact on the construction of stroke green channels and patient benefits. However, there is currently no teaching research in this area. Thus, in clinical practice, excellent technical skill alone is insufficient. Healthcare professionals must also possess strong communication abilities. Effective doctor-patient communication plays a critical role in whether treatment proceeds smoothly, and should be a major focus of medical education. In this study, we treated informed-consent conversations for IVT as a core module and one of the key teaching foci in stroke emergency training, systematically designing and intensively training this component.

The 5W1H analysis method is a systematic thinking tool widely used in business management, project management, daily life, and learning. By posing six questions-why, who, what, when, where, and how-it helps individuals systematically analyze and solve problems [25]. This study innovatively employed the 5W1H model for teaching, integrating theory with practice, integrating legal awareness, ethics, and professional knowledge throughout instruction, while emphasizing individualized informed-consent conversations. 

## Conclusions

This study introduced the 5W1H model into the informed consent process for IVT in AIS, integrating the elements of Why, Who, What, When, Where, and How throughout theoretical lectures, bedside learning, and scenario-based simulation teaching. The aim was to cultivate stroke green channel physicians who possess both advanced technical skills and humanistic competencies. Therefore, this research holds certain promotional value.

However, the study also has certain limitations: it is based on the accumulation of personal training experience and employs an observational research method without grouping the training subjects or conducting a statistical evaluation of the training effects. Consequently, more in-depth research is needed to further assess its effectiveness.
